# What influences antibiotic sales in rural Bangladesh? A drug dispensers’ perspective

**DOI:** 10.1186/s40545-020-00212-8

**Published:** 2020-06-03

**Authors:** Mohammad Abdul Matin, Wasif Ali Khan, Mohammad Mahbubul Karim, Sabeena Ahmed, Johannes John-Langba, Osman A. Sankoh, Margaret Gyapong, John Kinsman, Heiman Wertheim

**Affiliations:** 1 Infectious Diseases Division, icddr,b (International Centre for Diarrhoeal Disease Research, Bangladesh), Enteric and Respiratory Infections, 68 Shaheed Tajuddin Ahmed Sharani, Mohakhali, Dhaka, 1212 Bangladesh; 2 University of Kwazulu-Natal, School of Applied Sciences college of Humanities, Memorial Tower Building, Durban, F224 South Africa; 3 6INDEPTH Network, 38 & 40 Mensah Wood Street, East Legon, P. O. Box KD 213, Kanda Accra, Ghana; 4 Centre for Health Policy and Implementation Research, Institute for Health Research, University of Health and Allied Sciences, Ho, Volta Region Ghana; 5 Epidemiology and Global Health Unit, Department of Public Health and Clinical Medicine, Umeå University, SE-901 87 Umeå, Sweden; 6 Department of Public Health Sciences, Global Health (IHCAR), Karolinska Institutet, Stockholm, Sweden; 7 Department of Medical Microbiology and Radboudumc Center for Infectious Diseases, Radboudumc, Nijmegen, The Netherlands

**Keywords:** Antibiotics, Antibiotic dispensers, Pharmacy, Community, Drug regulation, Bangladesh

## Abstract

**Background:**

Antibiotic resistance poses a great threat to global health, especially in low- and middle-income countries with a high infectious disease burden and limited resources. In spite of regulations, antibiotics are sold in many settings as non-prescription medicines, resulting in inappropriate use and resistance.

**Objective:**

This study aimed to investigate the current status of access and use of antibiotics in rural Bangladesh, by exploring the perspectives and sales practices of antibiotic drug dispensers.

**Methods:**

We used a mixed methods approach (qualitative and quantitative). We mapped and characterized antibiotic purchasing and dispensing sites in the Matlab Health and Demographic Surveillance System catchment area. Furthermore, we investigated the volume of provision of systemic antibiotics in 10 drug outlets. We held 16 in-depth interviews with randomly selected antibiotics dispensers. Interviews explored factors associated with antibiotic selling. Responses were transcribed, coded for themes, and summarized. We used ATLAS.ti 5.2 for conducting a thematic analysis.

**Results:**

A total of 301 antibiotic dispensers were identified, of whom 92% (*n* = 278) were private and 8% (*n* = 23) public. 52% (*n* = 155) operated informally (i.e. without legal authorization). In order to promote and survive in their business, dispensers sell antibiotics for a range of conditions without a qualified physician’s prescription. Factors that facilitate these inappropriate sales include lack of access to healthcare in the rural community, inadequate doctor: population ratio, limited dispenser knowledge, poor pharmacovigilance concerning safety of self medication, lack of enforcement of policies, financial benefits for both customers and dispensers, and high dependency on pharmaceutical companies’ information.

**Conclusion:**

Dispensers in rural Bangladesh sell antibiotics inappropriately by ignoring existing national regulations. They operate the antibiotic sales without facing any legal barriers and primarily with a view to sustain their business, resulting in inappropriate sales of antibiotics to the rural community. The influence of the drug industry needs to be replaced with evidence-based, not commercially driven information. Awareness programs for antibiotic providers that promote understanding of antibiotics and antibiotic resistance through tailored interventions may be helpful in changing current antibiotic sales practices.

## Background

Inappropriate use of antibiotics in the community is common in many low- and middle-income countries (LMICs), where antibiotics are easily available without a prescription from retail drug shops [1, 2]. Major factors that influence overuse of antibiotics in LMICs include; imbalance of patient: health care worker ratio, absence of medical insurance, and an abundance of drug outlets selling antibiotics without prescription due to lack of enforcement of existing regulations [2–7]. Bangladesh has a relatively large generic drug manufacturing capacity [8, 9]. This has led to an abundance of local promotional activities by the pharmaceutical industry [8, 9]. For instance, retail drug outlets are visited on a regular basis by company representatives to provide antibiotics on credit or with economic incentives, resulting in overselling of antibiotics and other drugs irrespective of actual need [2, 8–14].

It is internationally well recognized that inappropriate and excessive use of antibiotics must be reduced to control antibacterial resistance, while maintaining access to these live saving drugs when they are needed [5, 14, 15]. One of the current priorities of the World Health Organization (WHO) is to raise awareness on prudent use of antibiotics and to reduce the incidence of infections that are resistant to antibiotics among the general public [5, 16, 17]. Since the promulgation of ‘Drug Control Ordinance’ in 1982 (which provided a regulatory basis for controlling the manufacturing, importation, distribution and sales of drugs), Bangladesh has made considerable progress in drug manufacturing while foreign multi-national pharmaceutical companies have lost market value in the country [9, 18, 19]. At present there are 1404 generic and 26,910 branded drugs available in Bangladesh, which meet 97% of the local demand while also facilitating a large export market to countries around the world [8, 9]. The monetary value of the volume of local production of all types of recognized drugs grew from US$21.6 million in 1981 to US$2.6 billion in 2017, and it is expected to reach US$3.4 billion by 2020 [6, 20, 21].

A significant proportions of antibiotics are sold without registered doctor’s prescription, which violates the National Drug Policy (NDP) 2005 and 2016 [6, 8, 9, 19, 22]. According to Directorate General of Drug Administration (DGDA) there are 119,751 registered retail pharmacy shops and nearly 300,000 informal (unlicensed) private pharmacies/drug store in Bangladesh [9, 18, 19]]. Most of these registered and unregistered pharmacies are run by individuals without formal training, and who may be working as insufficiently trained Pallichikishoks (village doctors) [6, 8, 19, 22]. Retail drug shops are the primary healthcare options for nearly 80% of the population for diagnosis (mostly clinic based) and treating common ailments [6, 8, 19, 22]. The care provided is based on experience and local common practices rather than evidence, with antibiotics being dispensed inappropriately as a result, contributing to antibiotic resistance in Bangladesh [6, 22–25].

There are several challenges that LMICs faces in their attempts to control antibiotic use. Just restricting the selling of antibiotics may have a negative consequence on the health system if no affordable and adequate alternative is provided. As a means of ensuring that antibiotic sales are only made to those who really need them, it is important to investigate the socio-cultural factors that influence the health care providers to sell these drugs.

## Objective

The aim of this study was to analyze the current status of antibiotics access and selling in rural Bangladesh (Matlab, HDSS) and to explore the antibiotic dispensers’ views, their actual practices and dispensing procedures as well as socio-cultural factors that influenced sales of antibiotics in this area. We intend to use the findings as the basis for developing a suitably tailored intervention program to improve the appropriate sales of antibiotics.

## Methods

### Study setting

This study is part of the multi-country ABACUS (Antibiotics Access and Use) study [26]. To understand how antibiotics are used in the communities in low and middle income countries, the study was conducted in six countries; two low income countries (Bangladesh and Mozambique), two lower middle income countries (Vietnam and Ghana), and two upper middle income countries (Thailand and South Africa). This study reports on our findings at the Matlab Health and Demographic Surveillance System (HDSS) of International Centre for Diarrheal Disease Research, Bangladesh (icddr,b) which was established in 1966. The Matlab HDSS maintains registration of births, deaths, and migrations, in addition conducts periodic census. It is situated on the bank of river Meghna, one of the main rivers of Bangladesh. The area includes 75 villages and 26,379 households. The population size of the study area is 111,259 of which 51,581 are males (46%) and 59,678 are females (54%).

We selected this site for the study as the household and population were all prelisted in the HDSS database, and access to the community is logistically straightforward, thereby facilitating the repeated visits that were necessary during the course of the study. We conducted field investigation from November 2016 to April 2017.

As detailed below, a mixed methods study design was followed, including an initial mapping exercise, followed by inventories conducted on the larger antibiotic dispensers. The high volume antibiotic dispensers (≥ 35 customers visit /day) includes 20 (7%) in the private and 1 (4%) in the public sector. However, in this study selected antibiotic inventories were conducted from those (*n* = 21) high volume dispensers proportionately 9 and 1 from private and public sectors respectively. This provided context on antibiotic selling practices. Qualitative interviews were also conducted with antibiotic dispensers.

### Mapping

We have initially mapped and characterized all kinds of formal (Drug license and Government authorization for drug selling) and informal (No drug license) antibiotic purchase and dispensing outlets from the entire Matlab HDSS government service area to discern antibiotic dispensers from different local markets and village shops. At first the study objectives were shared with the drug dispensers and a verbal consent was obtained. This was followed by registering the information on drug dispensing types; sources of financial support to run the business; average number of daily antibiotic encounters; drug outlet geolocation [by means of Global Positioning System (GPS) co-ordinates].

### Inventories

We performed inventories at the 10 largest outlets (identified during the mapping exercise as having the highest average daily number of customer encounters for antibiotic purchase). Written informed consent from the antibiotic dispensers was obtained. We assessed the presence of five essential systemic antibiotics [amoxicillin, amoxicillin-clavulanic acid, ciprofloxacin, trimethoprim-sulfamethoxazole, chloramphenicol] based on availability of antibiotics in 6 countries with different Gross Domestic Product (GDP). A digital thermo-hygrometer was used to assess the antibiotic’s storage temperature and humidity as measured at the actual storage site for each available brand of the particular antibiotic separately. We collected brand name, doses (mg) quantity, formulation, price in local currency, condition of blister pack, package insert, and expiration date. Structured questionnaires were used for both the mapping exercise; inventory and were administered to the formal and informal antibiotic dispensers.

### Qualitative tools and data collection

#### Sampling

A total of 16 in-depth interviews were conducted from 14 randomly assigned participants out of 278 private dispensers (retailers, diagnostic centre’s, grocery shops, village diarrhea treatment centre of HDSS) in addition, we interviewed two participants from 23 randomly assigned antibiotic dispensers representing the public sector (community clinics, union family welfare centers). Interviewees were sampled to ensure that all the geographical zones of the HDSS were included. We explored perceptions, reported practices, and social norms related to antibiotics selling using an open-ended, semi-structured interview guide. The interviews were classified into five sections: demographics, medicines (knowledge and dispensing practices), customer’s knowledge and awareness of antibiotics, antibiotic resistance, and regulatory issues. The data were collected by an anthropologist along with one sociologist, who had previous experience in qualitative methods of data collection. The anthropologist was trained for this work along with personnel from the other participating ABACUS sites, in order to ensure a consistent approach across all the sites with respect to the data collection process, data coding procedures, and data analysis. Interviews were audio recorded and field notes were also taken for subsequent clarification of any issues that arose, if needed. The duration of the interview was planned for 45–80 min. All questions, clarifications raised by the dispensers were resolved by the interviewer at the time of the interview. We conducted the antibiotics dispensers mapping from 15th November 2016 to 18th January 2017, and conducted the qualitative in-depth interviews from 15th March 2017 to 04th April 2017.

#### Data analysis

In-depth interviews were transcribed verbatim in Bengali, retaining all local terminology. We performed thematic content analysis, based on both a priori and inductively derived codes. A code sheet was developed by researchers based on the core themes of the questions, complemented by additional codes that emerged when reviewing the transcribed interviews to explicit imperative information. The analysis was facilitated by ATLAS.ti 5.2 software, on which we coded the data according to our research objectives. Though we analyzed each in-depth interview separately, in the results section we have drawn inferences collectively.

## Results

**Fig. 1:**
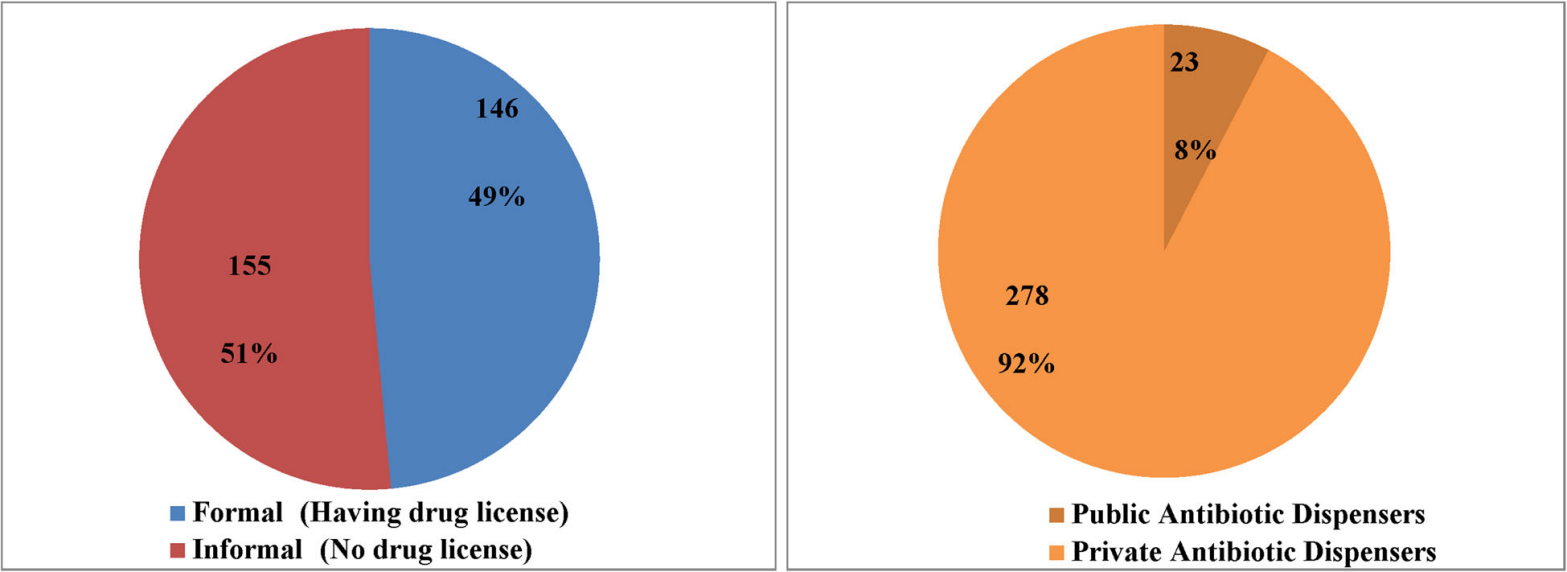
Status of antibiotic dispensers in the study area

In total 301 antibiotic dispensers were identified from our study area. This includes both private (*n* = 278; retail drug store, diagnostic center, hospital pharmacy, grocery shops) and public facilities (*n* = 23; Community Clinics, Union Family Welfare Centers). Out of 278 private drug shops 155 (51%) had no formal authorization for selling antibiotics (Fig. 1). These unlicensed shops were run by untrained or undertrained village doctors and drug dispensers. Most frequently dispensed antibiotics from those outlets were azithromycin, cefixime, cephradine, ciprofloxacin, amoxicillin, flucloxacillin, and ceftriaxone.

Among the 10 high volume customer visit (*n* ≥ 35) drug dispensing outlets none had all five essential antibiotics. However, drug inventory conducted on those 10 outlets we found 4 out of 9 (44%) in private and in 1 public sector - 4 and 2 essential antibiotics respectively. Amoxicillin, Amoxicillin+Clavulanic Acid and Ciprofloxacin were widely available while Co-trimoxazole was rarely found in private drug outlets. Chloramphenicol (oral and injectable formulations) was not present either in private or public settings. However, Co-trimoxazole and Amoxicillin were frequently present in the public sector, whereas Ciprofloxacin, Amoxicillin and Clavulanic Acid were not.

**Fig. 2:**
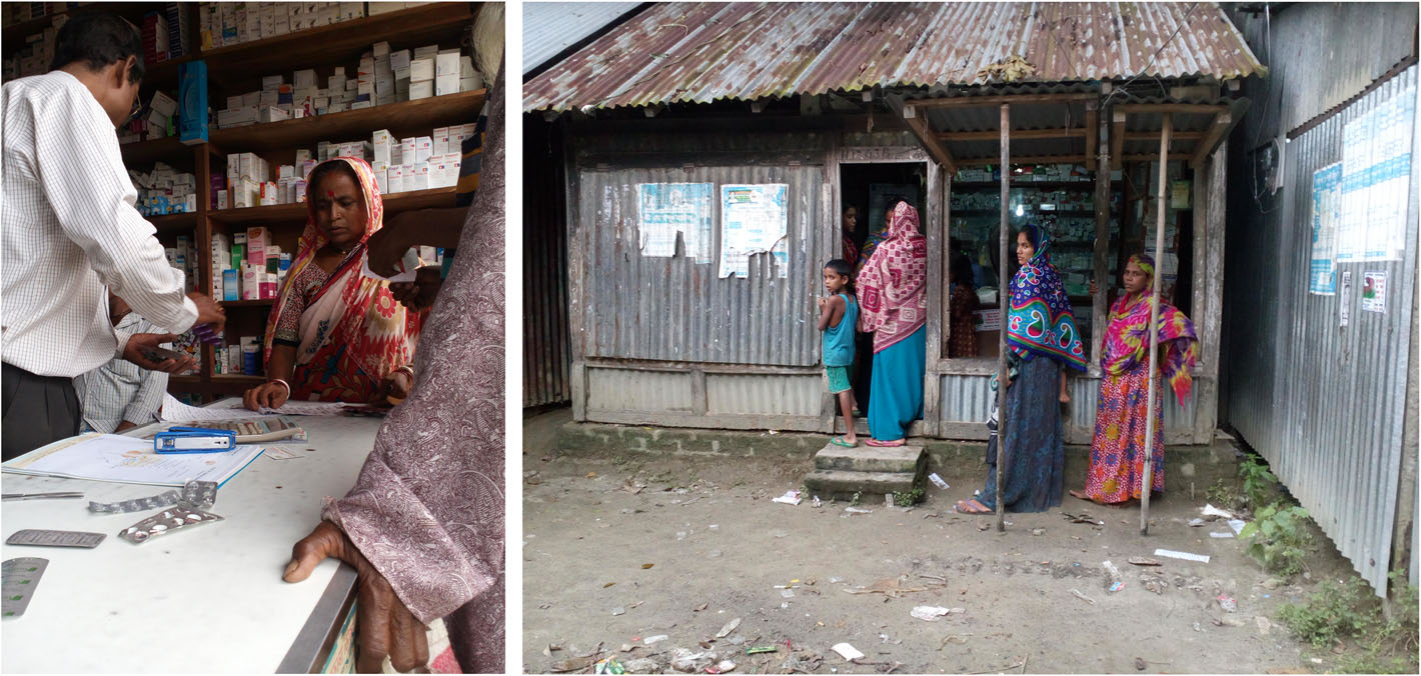
Formal and informal drug outlets

When assessing the storage conditions of public and private outlets, antibiotics were found stored in various inappropriate locations such as on the floor (Fig. 2); inside the drug dispensing shops the temperature varied between 22.0 °C to 28.8 °C and the humidity 43 to 65%. The mean storage temperature in private drug outlets was higher (25.6 °C) compared to public drug dispensing settings (22.0 °C) that was measured during winter season. However, we know for instance amoxicillin need to be stored between 15°and 25 °C, should be protected from light and moisture, ciprofloxacin at 21 °C to 24 °C (room temperature), Sulfamethoxazole-trimethoprim at ≤24 °C.

There were several brands of each antibiotic found in most of the drug stores. For instance; ciprofloxacin (16 different brands): amoxicillin (8 brands) and amoxicillin-clavulanic acid (5 brands). Having such high number of brands not only confuses the customers but also for survival in this competitive market the Pharmaceutical companies offer different sorts of promotional activities to doctors as well as the drug dispensers.

### In depth interviews

We conducted in-depth interviews with 16 dispensers, twelve of whom were both owner and seller. Ten of the 16 informants were aged from 20 to 40 years, and the remaining were aged 41 to 60 years old. They had completed secondary (5), higher secondary (8), graduate (1), and post graduate (2) education. Among them there were retailers (13), government health care providers (2), and grocery shop keeper (1). Three informants were female and the rest (13) were male.

### Themes

The findings from the qualitative interviews are presented within the following themes: i) Selling practices; ii) Sources of knowledge about medicine and the influence of promotional activities; iii) Knowledge about antibiotic use and antibiotic resistance; iv) Selling incomplete courses of antibiotics; v) Selling practices of branded antibiotics; vi) Dispensers’ instructions provided with dispensed medicines; vii) Enforcement of rules and regulations; *viii)* What steps should be taken to improve use of antibiotics?

#### i) Selling practices

All (*n* = 16) the informants narrated that they can run their business without facing any legal barriers. Medicines, including antibiotics, are generally dispensed without a physician prescription from their outlets. According to the respondents, antibiotics are sold based on the health concerns and visible symptom of the customers. Additionally, they also get specific requests for an antibiotic when shown an empty blister pack by the customer or they mention a preferred brand name. Dispensers provide the drugs that they expect the patient wants and needs. If they do not provide medicines according to the customer demand, they will leave the shop and go to another seller and this will adversely affect their business. Informants stated that most of the people ask for antibiotics without prescription. Young people reportedly come to buy medicines without any doctor’s consultation comparatively more often than elderly people.

One respondent mentioned, “*Customers buy medicines along with antibiotics without physician prescription and they know well that if they consume antibiotics, they will be cured from illness very easily. Thus they want to consume antibiotics for simple illness without a doctor’s prescription.”* (Informant- Age: 38, Sex: Male, facility: private business)

Another informant noted, *“Some customer purchases medicine two times with a prescription, and later they have a tendency to buy medicines without prescriptions based on previous experience.”* (Informant- Age: 35, Sex: Male, facility: private business)

Dispensers pointed out that they generally accept returned unused medicines, including antibiotics, from known clients. Customers consider antibiotics are costly medicines compared to others, and they prefer to return leftover antibiotics to dispensers. They are inclined to accept their sold medicines back after checking the blister pack condition and expiry date. In case they are in an acceptable condition, they exchange them for other medicines or refund, as a means to maintain good relationship with the customers. Thus, they resell returned drugs to other customers without any financial loss.

One informant narrated, *“Sometimes people come to return sold medicines, including antibiotics. I have no option other than taking these back and I have to return the money to maintain good relationship with the customers.”* (Informant-Age: 46, Sex: Male, facility: private business)

Another informant stated, *“Based on the good terms I have with a customer, I sometimes take back the sold Ciprofloxacin because the customer pretends to me that they have been fully cured from the disease.”* (Informant-Age: 38, Sex: Male, facility: private business)

A third said, *“I have to take back sold medicines from customers, otherwise they will be disappointed.”* (Informant-Age: 58, Sex: Male, facility: private business)

#### ii) Sources of knowledge about medicine, and the influences of promotional activities

Informants stated that often they start their pharmaceutical business with little knowledge about medicines. Their source of information is primarily from the representatives of the pharmaceutical industry. They usually acquire their knowledge about the medicines from drug information leaflets, brochures, treatment guidelines and medicine directories provided by different pharmaceuticals companies. They also receive different types of financial incentives from the industry, such as special discounts to sell a particular product, financial incentives, gift boxes, and invitations to attend commercial medical education sessions. They are also informed about new medicines through pharmaceutical representatives, and through the internet websites of different pharmaceutical companies. The informants also become acquainted with medicines by observing what physicians do for various clinical syndromes. They also learn about medicine from short course training provided by the Ministry of Health & Family Welfare (MoFW), and pharmacist training. Further, they may participate in dissemination meetings or seminars arranged by pharmaceutical companies with the aim to increase their sale.

One informant narrated, *“I gathered knowledge about medicine from my 8 years’ job experience in medicines shop. At that time, I asked other colleagues about application of prescribing drug. I also learned about selling medicines from sales representative.”* (Informant- Age: 38, Sex: Male, facility: private business)

Another respondent stated, *“It is difficult to get an idea about all medicines nevertheless I have followed working experience with this business and observe how doctors prescribe.”* (Informant- Age: 35, Sex: Male, facility: private business)

The informants stated the influence of pharmaceutical representatives and advertisers contribute to inappropriate sales of antibiotics. Pharmaceutical companies regularly arrange dissemination meetings, conferences, and seminars of their products for local drug dispensers in order to increase their sales in the rural markets. Area managers and medical representatives conduct these sessions. Retail drug outlets are visited on a daily basis to get drug orders by company representatives. They influence dispensers to make larger orders of their medicines with antibiotics: for example, if they purchase five boxes of a certain drug then the additional one box of medicine and antibiotic is given free; or they may receive monthly credit in order to achieve their selling target.

#### iii), Knowledge about antibiotic use and antibiotic resistance

Informants stated that antibiotics are effective for different diseases and it helps people to recover quickly from their illness. They often use antibiotics to cure high fever, cold, cough, acute watery diarrhea, skin disease, accidental injuries to cure wound and infections, and any kind of post-surgical infections that are not cured by common drugs. Drug sellers are repeatedly recommended by the pharmaceutical companies antibiotics like Azithromycin for high fever, respiratory tract infections (RTI), skin and soft tissue infections. Antibiotics help in ‘preventing ability’ inside the body to protect it from germs. Informants also explained that beside benefit from antibiotics also causes side effects for instance dizziness, feeling weak, diarrhea, vertigo, and allergy.

One respondent pointed out, *“I observed antibiotics are recommended by other colleagues of our drug store e.g. Ciprofloxacin, when high fever is not managed by paracetamol.”* (Informant- Age: 20, Sex: Male, facility: private business)

Another informant said, “*I have no profound insights about antibiotic use and antibiotic resistance, so what I do is to copy how doctors prescribe antibiotics.”* (Informant- Age: 58, Sex: Male, facility: private business)

One drug seller mentioned, *“In my consideration, paracetamol works for fever, and antibiotics work to add to the strength of the body to kill the germ.”* (Informant- Age: 55, Sex: Male, facility: grocery shop)

Another drug seller narrated his knowledge on antibiotic use as *“Antibiotics are the medicines which are used for 6-7 types of diseases.”* (Informant- Age: 46, Sex: Male, facility: private retailer)

When asked about the meaning of the term “antibiotic resistance”, the dispensers had varied responses. They mentioned that if the wrong doses of antibiotics are prescribed for different illnesses, these antibiotics may not work in the human body if used for a long time. According to another informant’s observation, if antibiotics are not completed according to the prescribed durations, it will not able to kill the germ, the germ will persist in patient’s body and they will be affected by the same disease again, then the antibiotics need to be changed, and stronger antibiotics are needed that are expensive, and the treatment time will become longer.

Informants also narrated that when first and second line antibiotics are not effective then they recommend third or fourth line such as Cephalosporins or Quinolones. Due to the availability of these drugs and for higher profit margin the drug sellers are also inclined for marketing these antibiotics.

One informant stated, *“Many customers do not consume all dispensed antibiotic pills when they feel better from illness, and when they are affected again after a few days, then they consume the remaining antibiotic for a second time but now they don’t feel cured. This time that antibiotic is not working in their body and this situation is called antibiotic resistance.”* (Informant- Age: 42, Sex: Male, facility: private business)

Another informant said, “*My customer alleges that antibiotics have a strong role to recover illness quickly and on the other hand it shows some toxicities i.e., weakness, loss of appetite, affects liver & kidney. But I have no clear idea if it has any association with antibiotic resistance or not.”* (Informant- Age: 40, Sex: Male, facility: private business)

#### iv) Selling incomplete courses of antibiotics

Informants cited that they regularly sell incomplete courses of antibiotics to their customers, as customers have limited financial capacity. They stated that first people want to start with minimal dosage and if they see good response are hesitant to go for remaining dosage of antibiotics. In that case they do not need to spend a large amount of money at one time. Informants also sell incomplete doses of regimens for maintaining good relation with familiar customers, and thereby helping to ensure that the customer comes back again in future to buy antibiotics and other medicines.

One informant stated, “*I have to sell incomplete doses of antibiotics to maintain good relationship with the customers. If I don’t sell incomplete doses then customers will purchase from another drug store, as a result I will lose the customers and income, so for surviving with the business I sell incomplete doses of antibiotics.”* (Informant- Age: 48 Sex: Male, facility: private business)

Another informant said, *“It is a village area, people are not financially solvent. I suggest and sell them antibiotics for 3 or 4 days. Meanwhile, if they feel better, they do not want to continue even for these 3 to 4 days, and they stop sooner.”* (Informant-Age: 56, Sex: Male, facility: private business)

Another respondent stated, *“I saw many people do not follow doctors’ recommendation regarding antibiotics. Suppose someone needs to take a seven days course, but s/he buy it for three days, and after three days if s/he recovered then s/he does not buy the rest.”* (Informant- Age: 48, Sex: Male, facility: private business)

Informants said that they usually recommend antibiotic doses based on age, body weight and severity of illness, as well as their own experience. They provide antibiotics for upper respiratory tract infections which are often viral, and for which they should not be providing antibiotics. They stated that they do not have good knowledge of the correct dose. Further, their selection of the recommended duration of treatment varies and is inconsistent: some of them prefer to recommend a 7-days course, and others suggesting a 3 to 5-day course for the same illness.

One informant explained, *“If a patient suffers from fever, I ask the patient about how long they suffer from fever. If customers suffer for 3-4 days, then I give an antibiotic; if shorter duration of fever I give them paracetamol only. I suggest customer to consume antibiotics 7 days or at least 5 days.”* (Informant- Age 38, Sex: Male, facility: private business)

#### v) Selling practices of branded antibiotics

Dispensers mentioned that certain customers want to purchase a specific brand or type of antibiotics for their illness without a doctor’s prescription. Sometimes they are unable to recall the exact brand name, but customers express their requirements based on certain colors (capsules red and tablets white), size & shape.

As a result, Amoxicillin, Ciprofloxacin, and Azithromycin are sold on customers’ specific request. Because customers sometimes show a preference about their choices regarding certain brand of antibiotics, dispensers respect customer desire to ensure that they come again in future so that they do not lose the customer.

One respondent mentioned, “*If a customer requests a specific antibiotic I provide the same. However, if it is not available, I advise to take an alternative antibiotic but sometimes they do not express their interest to buy that medicine.”* (Informant- Age: 46, Sex: Male, facility: private business)

#### vi) Dispensers’ instructions provided with dispensed medicines

**Fig. 3:**
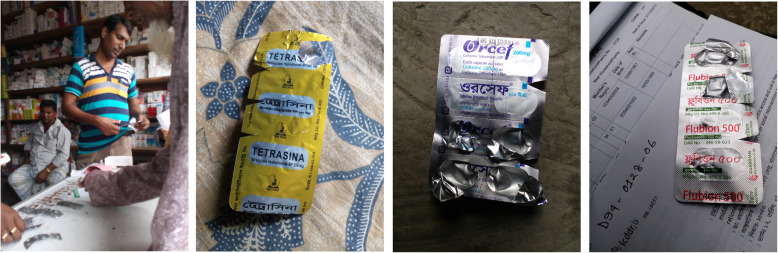
Techniques to remember antibiotic dosage taken by cutting corners of the antibiotic strips

All informants reported they provide instructions to their customers about the dosage of the drugs they sell and the duration of its use. Informants mentioned they usually provide oral and written instruction to the customers. Sometimes they cut corners of the strip with numbers that coincide with the number of dosage they need to take per day as a way to remember, in particular for customers who are unable to read or write (Fig. 3).

The drug seller cuts the medicine strip edges for the uneducated customer to remember daily dose schedule. Medicines sold by the dispensers to the customer are instructed on the number of pills to be taken daily, as well as the duration, verbally and/or written on a piece of paper. Some of the dispensers mention they also write instructions on the package.

Dispensers suggest people to complete the antibiotic course according to the recommendations; otherwise the drugs will not work next time. But customers reportedly do not always take this advice. It was observed that the dispensers do not inform the customers about possible adverse effects of antibiotics as some drugs can cause nausea, rash, vomiting, diarrhea, and poor appetite. These side effects result in customers to stop the course prematurely. Additionally, customers do not always share information with the dispensers about previous allergic reactions to, for example, penicillins, and thus they may purchase them again.

One informant explained, *“Customers are not giving importance to my words. Sometimes I don’t want to give the instruction to customers because they consulted with a doctor or relatives who are more experienced compare to drug sellers like us.”* (Informant-Age 38, Sex: Male, facility: private business)

Another seller narrated, *“I usually give prescription and instructions to the customer in relation to consuming medicines. I explain to the rural women that they should complete full course of antibiotics, but they don’t understand.”* (Informant-Age 56, Sex: Male, facility: private retailer)

Informant stated, *“I don’t bother asking to the customer about diseases as it is not my business. My responsibility is only selling medicines, not to provide instructions to the customers. I give verbal instructions to the customers because I am not a doctor, so I can’t give written instructions.”* (Informant-Age 35, Sex: Male, facility: private business).

#### vii) Enforcement of rules and regulations

There are specific guidelines on how to prescribe or dispense antibiotics in Bangladesh. However, in rural areas where there is often no access to expertise and diagnostic tools it can be challenging to follow these guidelines. Dispensers stated that the “Drug Inspector” is empowered by the Government to act as the Licensing Authority of drugs for the purpose of issuing licenses to manufacture, store, sell, import and export drugs and medicines. Due to shortage of manpower within the Directorate General of Drug Administration, it is not possible for them to inspect all pharmaceutical dispensers at regular intervals. Informants also cited that they do not ever see any Government authority taking the initiative to enforce the existing rules and regulations, or concerning any prohibition or penalty for selling antibiotics without prescription. According to the conditions of receiving a drug license, medicines should be dispensed by registered pharmacists, but in reality no registered pharmacist is found in most of the drug stores. Informants added that they do not ever face any legal difficulty to run their pharmacy business, selling medicines and antibiotics without prescription. If the government would strictly enforce the regulations that require antibiotics to be sold only with a registered doctor’s prescription, dispensers would not sell antibiotics without prescription and thus there would be less chance of developing antibiotic resistance. However, this would also create access challenges for people who really need the medicines.

One respondent mentioned, *“Recently, the High Court of Bangladesh ordered that prescriptions should be either printed or handwritten in capital letters. However, this is not followed in most instances. Yesterday, I saw one prescription which I didn’t understand. If we obey the rules and regulations and follow in all cases, then the inappropriate selling of antibiotics in Bangladesh will be changed.”* (Informant-Age: 35, Sex: Male, facility: private business)

Another narrated, *“I think some pharmacy store-keepers know the rules of selling drugs and some don’t, however, none fall the regulations strictly. They just sell and want to make profit by selling medicine to the customers”* (Informant-Age: 38, Sex: Male, facility: private business)

Informants stated, “*I sell a variety of different grocer products (rice, oil, biscuit, and spices) including some drugs along with antibiotics from here. I am now doing this business to support my son’s education expenses; actually, I have no idea about rules and regulations of our country about selling medicines. Nobody informs me about this. I just sell different medicines with a view to help the local people, because they ask medicines from me as well.”* (Informant-Age: 58, Sex: Male, facility: private business)

#### viii) What steps should be taken to improve use of antibiotics?

Informants suggested several steps that should be taken to improve the appropriate sales of antibiotics:

- Provide training to pharmacy drug storekeepers and village doctors on correct procedures for ensuring appropriate antibiotic sales.
- Conduct awareness campaigns for community members in order to raise knowledge on the proper use of medicines and antibiotics within the community. Print and electronic media can play an important role in disseminating information among the population.
- Engage local leaders and key institutions - for example, members of parliament, concerned ministries, and regulatory authorities - in awareness campaigns to make them effective. Such awareness programs need to be conducted on an ongoing basis in order to ensure that people will learn and then change their current practice.
- Instead of consuming antibiotics, use traditional medicine for upper respiratory tract infections, like ‘*TULSI LEAF*.’ Herbal medicines can be an acceptable and also effective alternative to treat cough and cold.

## Discussion

This study investigated antibiotic dispensers’ perspectives on antibiotic access and use in a rural setting of Bangladesh, both licensed and unauthorized private drug sellers as well as public health facilities participated in the research. Although the National Drug Policy (2005 and 2016) prohibits antibiotic sales without a prescription from a qualified doctor, this is ignored by the dispensers due to lack of monitoring by the drug regulatory authorities, financial incentives, and limited awareness and knowledge of the risks of unnecessary antibiotic use.

This study reveals a high degree of inappropriate antibiotic dispensing in the study area, in terms of both selling antibiotics without a proper diagnosis or a prescription, and the provision of incomplete antibiotic regimes in terms of dose or duration. Financial constraints are the major impediment to health-seeking behavior from formal health care facilities. Patients prefer to go to drug outlets where they can get the medicines just by explaining their health problems, without any laboratory diagnosis or a prescription. This results in antibiotics being sold without proper instructions, and with an increased likelihood that they will have an incorrect dose and treatment duration. Interestingly we also found that unused drugs are taken back by the drug sellers and resold, which may be an important health risk as the condition of the drug is uncertain. This is not considered good pharmacy practice.

Inappropriate antibiotic use and widespread selling without physician prescription is common in many LMICs [27–29]. But this is also common practice in some higher income countries like Spain and in eastern and southern Europe [5, 29]. Non-prescription sales of antibiotic is due to a complex interplay of various drivers, like: knowledge of antibiotics and infectious diseases by dispenser and customer, financial issues of both drug dispensers and consumers, paucity of treatment guidelines for antimicrobial use, and lack of enforcement of the regulations [27–29]. To break this pattern we need to focus on multifaceted and multilevel, tailored interventions that are sustainable without too much additional costs [27]. A study conducted in Thailand to promote and sustain good prescription practices especially with regards to social norms was an important achievement, which subsequently promoted nationwide expansion. The program is organized on two levels: a network of multidisciplinary groups (i.e. local partners) at the health-care delivery level, and in subsequent this team collaborated with policy-makers, academics and researchers from national health agencies and universities to form central partners [30].

In our study area, 155 out of 278 (51%) unlicensed retail drug shops are running where antibiotics are sold by untrained or poorly trained drug store owners. We did not identify any graduate pharmacists in any of the 301 antibiotic dispensing outlets that we located. Most of the drug shops are run by individual owners with limited expertise, which leads, for example, to improper storage of drugs. This is a violation of Bangladesh’s drug policies 2005 and 2016. There is no one to monitor or enforce this because of the limited work force of the DGDA [6, 8]. This makes an opportunity for the drug industry to become the main source of information for those sellers [8, 9]. Selling inappropriate course of antibiotic or shortened treatment regimen for instance a day or two is observed quite frequently in the study area by the drug dispensers which is very critical to human rights, code of ethics and the health section. Their primary interest is to sell drugs, including antibiotics, and to make profits. Absence of qualified sellers in pharmacies has also been reported in Vietnam, Sri Lanka, India and Pakistan [1]. It was seen in Palestine that community pharmacy operations were more business-oriented than health services [1], so the findings from our study corroborate what has previously been found elsewhere in South and South East Asia .

The sellers are influenced by promotional offers of the pharmaceutical companies to sell more medicines, including antibiotics [22, 23, 25]. The pharmaceutical companies are also their main source of information. The drug sellers are deeply embedded in the local community and culture, and they provide low-priced services and are, for many, the first point of contact for health issues [23]. They regularly dispense incomplete courses of antibiotics to financially comprised customers instead of recommended courses, in order to maintain a good relation with them for future business [22, 23, 25]. Client simulations studies showed that 78% of pharmacies in Saudi Arabia, 87% in Syria, and 94% in India sell antibiotics without a prescription, a percentage that increases further when the client simulators insisted on buying antibiotics [2]. It was revealed from the study that the most frequent reason for buying antibiotics was acute upper respiratory tract infections, which are usually self-limiting [2] Additionally, due to lack of good diagnostic facilities in the rural Bangladesh, the pharmaceutical representatives take further advantage to train the shop owners how to treat empirically rather than utilizing an evidence based when selling their drugs [22, 23, 25]..

The reported high percentage of non-prescribed sales of antibiotics is worrisome, given its association with unnecessary use, too short courses and inappropriate drug and dose choice, and subsequently, the development of antibiotic resistance [31]. We found that the drug sellers had some knowledge about antibiotics but were not aware about the term “Antibiotic Resistance”. Both sellers and patients believe that antibiotics facilitate prompt recovery from illness, and therefore rely on antibiotics as compared to others drugs [22, 23, 25]. Therefore access to antibiotics for patient is considered to fill in a perceived need and therefore it will be challenging to change the behavior [23, 27].

Regulation is crucial to safeguard access to antibiotics, but a transition towards such regulation needs governmental commitment and improvements in health systems that are not possible in many countries [27]. Routine monitoring (inspection and checking process) of the local drug dispensers outlets by the drug regulatory authorities appears to be superficial and is hardly ever done in the rural settings like Matlab [18, 23]. We did not detect any penalties given to sellers for selling antibiotics without following a qualified doctor’s prescription. In the study areas antibiotics are even available in some grocery shops where no monitoring will ever be conducted. As there is a lack of enforcement of the regulations, self-medication is possible, and this is viewed as more economical and convenient by many customers than consulting a health professional [2]. Thus routine monitoring / inspection of drug regulatory authorities is essential to improve the situation in combination with public awareness campaigns. Studies from Vietnam, Laos and Palestine highlighted the need for the enforcement of regulations at community pharmacies [1]. Ban of over-the-counter sale of antibiotics has been implemented in Chile with the aims of improving optimal use of antibiotic among consumers and prescribers [32]. Other evidence also suggests the need for the training of dispensers as well as improvement in dispensing practices in community settings [1]. Evidence suggests that if the sellers take a few minutes to consult the patients about the importance of completing the dose, this could be an effective intervention. Direct education of patients also improved antibiotic compliance and decreased use of unrestrained drugs in Peru [33] Concerted and consistent efforts can successfully educate the community people that antibiotics are a precious and finite natural resource that should be conserved rather it should not be used to treat viral infections [34, 35]. This will allow patient-centered decision making about antibiotic treatment, where patients and doctors can balance confidence that a complete and lasting cure will be achieved against a desire to minimize antibiotic exposure [32, 34]. Reducing unnecessary antibiotic use is therefore essential to mitigate antibiotic resistance [34, 35]. Dissemination of, Education and training incentives to health-care providers such as prescribers and dispensers in the community have been recommended on appropriate use of antibiotics to contain resistance [33]. A program on optimal use of antibiotic is to be adjusted to local context to help with treatment decisions and to help the community pharmacists make good decisions about antibiotic dispensing [36]. More such studies are needed on how to balance the effects of specific interventions on individual health versus increasing the resistant bacteria population [27–29].

### Strengths and limitations of the study

This study is part of a multi- centered observational study. As the study was conducted in rural setup of Bangladesh, we expect that the findings will be relevant to antibiotics sales in other socially and culturally similar areas of Bangladesh. Although there are number of antibiotics widely available in this study area, we considered five essential antibiotics in order to compare data with other sites, and thus we may have missed some locally relevant antibiotics. We selected informants for in-depth interview proportionately from three hundred and one located private and public drug outlets, this has likely imbalanced the ratio of interview between them. Since interviews of the drug dispensers were only possible when the shops were open at the same time customers also visit this might have interrupt the flow of discussion.

## Conclusions

The findings from this study indicate that sellers are heavily influenced by the drug industry to sell antibiotics despite the customers are not always in need. However, the drug authority need to enforce on drug sellers that drugs like fluroquinolone not to recommend as first line therapy for uncomplicated urinary tract infections due to the development of resistance specially *Escherichia coli* [37, 38].

The dispensers are not sufficiently trained for proper dispensing of antibiotics, which results in poor instructions provided to customers and also the potentially risky practice of taking back leftover sold drugs for future sell. All these factors result in widespread misuse of antibiotic in the study area, and consequently the emergence of antibiotic resistance. Improving this situation would require; strengthening the regulatory process by the Directorate General of Drug Administration Bangladesh, close supervision by the relevant authority, provision of reliable information to drug sellers that is independent of the pharmaceutical industry, and promoting awareness programs both for the community and for drug dispensers to increase understanding of antibiotic resistance through effective communication, education and training.

## Supplementary information

**Additional file 1.** Appendix A. eCRF registry form drug dispenser characteristics (for mapping exercise). Appendix B. eCRF_Dispensers Inventory. Appendix C. Preparatory_Antibiotic Dispensers_In-Depth Interview Guide.

## Data Availability

The supporting data of the results or analyses can be made available from the corresponding author on reasonable request.

## Abbreviations

INDEPTH: International Network for Demographic Evaluation of Population and their Health in Low and middle Income Countries
icddr,b: International Centre for Diarrheal Disease Research of Bangladesh
HDSS: Health and Demographic Surveillances System,
FRA: Field research assistant
GPS: Global positioning system
GDP: Gross domestic product
FGD: Focus group discussion
IDI: In depth interview
ICF: Inform consent form
ABACUS: Antibiotic access and use

## References

[1] Azhar Hussain and Mohamed Imam Ibrahim, Perceptions of Dispensers Regarding Dispensing Practices in Pakistan: A Qualitative Study Tropical Journal of Pharmaceutical Research, 2011. 10, (2) 117–123.

[2] Thuy NDT (2014). Antibiotic sales in rural and urban pharmacies in northern Vietnam: an observational study. BMC Pharmacol Toxicol.

[3] Alsan Marcella, Schoemaker Lena, Eggleston Karen, Kammili Nagamani, Kolli Prasanthi, Bhattacharya Jay (2015). Out-of-pocket health expenditures and antimicrobial resistance in low-income and middle-income countries: an economic analysis. The Lancet Infectious Diseases.

[4] Nina V, et al. ‘Practical knowledge’ and perceptions of antibiotics and antibiotic resistance among drugsellers in Tanzanian private drugstores. BMC Infect Dis. 2010;10(1):270–78. 10.1186/1471-2334-10-270 PMC2949758 20846407

[5] Daniel M (2011). Non-prescription antimicrobial use worldwide: a systematic review. Lancet Infect Dis.

[6] Shill Manik Chandra, et al., Medication Practices in Bangladesh, Roles of Pharmacists at Current Circumstances International Journal of Pharmacy and Pharmaceutical Sciences 2011. 3 (4), 5–8.

[7] Mayadah S (2012). Knowledge, attitudes and behavior regarding antibiotics use and misuse among adults in the community of Jordan. A pilot study. Saudi Pharmceutical J.

[8] Khan Mohammad Shahidul Islam. National Drug Policy 2016 (English Version) 2016. Last update date, 21 September 2017, [cited 2016 December 19].

[9] Mostofa Musa, et al., Antibiotic Use and Resistance in Bangladesh : Situation Analysis and Recomendation in Global Antibiotic Resistance Partnership- Bangladesh (GARP), Bangladesh National Working Group. Antibiotic Use and Resistance in Bangladesh : Situation Analysis and Recomendation , Centre for Disease Dynamics, Economics & Policies (CDDEP) Washington, DC and New Delhi. January 2018, Directorate General of Drug Administration, Mohakhali, Dhaka-1212: GARP- Bangladesh Secretariat.

[10] Abdulhak AB, et al. Non prescribed sale of antibiotics in Riyadh, Saudi Arabia: A Cross Sectional Study. BMC Public Health. 2011;11(1):538–42. 10.1186/1471-2458-11-538 PMC3146870 21736711

[11] Gebremedhin Beedemariam Gebretekle, Serbessa MK. Exploration of over the counter sales of antibiotics in community pharmacies of Addis Ababa, Ethiopia: pharmacy professionals’ perspective. Antimicrobial Resist Infect Control. 2016;5(1):2–8. 10.1186/s13756-016-0101-z PMC4734870 26835006

[12] Biswas M, et al. Self medicated antibiotics in Bangladesh: a cross sectional health survey conducted in Rajshahi city. BMC Public Health. 2014;14(1):847–53. 10.1186/1471-2458-14-847 PMC4150958 25124712

[13] Dillip A, et al. What motivates antibiotic dispensing in accredited drug dispensing outlets in Tanzania ? A qualitative study. Antimicrobial Resist Infect Control. 2015;4(1):30–37. 10.1186/s13756-015-0073-4 PMC4509560 26199723

[14] Hawking Meredith KD, Lecky Donna M, Touboul Lundgren Pia, Aldigs Eman, Abdulmajed Hind, Ioannidou Eleni, Paraskeva-Hadjichambi Demetra, Khouri Pauline, Gal Micaela, Hadjichambis Andreas Ch., Mappouras Demetrios, McNulty Cliodna AM (2017). Attitudes and behaviours of adolescents towards antibiotics and self-care for respiratory tract infections: a qualitative study. BMJ Open.

[15] Bithi SS (2014). Drug utilization study in orthopaedic units: Antibiotics prescribed in hospital out-patients in Dhaka, Bangladesh. Int Curr Pharm J.

[16] Chandy S (2013). Patterns of antibiotic use in the community and challenges of antibiotic surveillance in a lower-middle-income country setting: a repeated cross-sectional study in Vellore, South India. J Antimicrob Chemother.

[17] Barker A (2017). What drives inappropriate antibiotic dispensing? A mixed-methods study of pharmacy employee perspectives in Haryana, India. BMJ Open.

[18] The Drugs (Control) Ordinance 1982. Directorate general drug administration (DGDA), 2017. Law and Parliamentary Affairs Division of the People’s Republic of Bangladesh http://www.dgda.gov.bd.

[19] Parvez Morshed Chowdhury, et al., An overview of the pharmaceutical sector in Bangladesh. 2010, BRAC Research,: BRAC Stock brokerage Limited. https://www.scribd.com/doc/315428766/BD-Pharma-Overview-2010-BRAC.

[20] Local Production of Pharmaceuticals and Related Technology Transfer in Developing Countries: , in A series of case studies by the UNCTAD Secretariat. 2011: Essential medicines and health products information portal a World Health Organization resource. 320.

[21] Tashnim, Mohammad. Bangladesh Pharmaceutical Industry 101 2017. published date 27 July 2017.

[22] Ahmed SM, Hossain MA (2007). Knowledge and practice of unqualified and semi-qualified allopathic providers in rural Bangladesh: implications for the Human Resources for Health problem. Health Policy.

[23] Ahmed SM, et al. Exploring the status of retail private drug shops in Bangladesh and action points for developing an accredited drug shop model: a facility based cross-sectional study. J Pharm Policy Pract. 2017;10(21). 10.1186/s40545-017-0108-8 PMC5506600 28702204

[24] Chowdhury FR (2006). Rationality of drug uses: its Bangladeshi perspectives. Mymensingh Med J.

[25] Jiben R (1997). Health status, treatment and drug use in rural Bangladesh: a case study of a village. Australian J Rural Health.

[26] Wertheim Heiman F.L., Chuc Nguyen Thi Kim, Punpuing Sureeporn, Khan Wasif Ali, Gyapong Margaret, Asante Kwaku Poku, Munguambe Khatia, Gómez-Olivé F. Xavier, Ariana Proochista, John-Langba Johannes, Sigauque Betuel, Toan Tran Khanh, Tollman Stephen, Cremers Amelieke J.H., Do Nga T.T., Nadjm Behzad, van Doorn H. Rogier, Kinsman John, Sankoh Osman (2017). Community-level antibiotic access and use (ABACUS) in low- and middle-income countries: Finding targets for social interventions to improve appropriate antimicrobial use – an observational multi-centre study. Wellcome Open Research.

[27] Laxminarayan R (2013). Antibiotic resistance : the need for global solutions. Lancet Infect Dis Comm.

[28] Carlet J (2011). Society’s failure to protect a precious resource: antibiotics. Lancet Infect Dis.

[29] Afari-Asiedu S (2018). To sell or not to sell; the differences between regulatory and community demands regarding access to antibiotics in rural Ghana. J Pharm Policy Pract.

[30] Sumpradit N (2012). Antibiotics Smart Use: a workable model for promoting the rational use of medicines in Thailand. Bull World Health Organ.

[31] Laura Maria Francisca Kuijpers, et al., Enteric Fever in Cambodia: Community Perceptions and Practices Concerning Disease Transmission and Treatment. Am Soc Trop Med Hygiene, 2018. 00, (0) 1–9. 10.4269/ajtmh.18-0432 PMC6283521 30298811

[32] Veronika W, Anahí D, Gonzales R (2010). Trends in antibiotic utilization in eight Latin American countries, 1997–2007. Rev Panam Salud Publica.

[33] Okeke Iruka N, Klugman Keith P, Bhutta Zulfiqar A, Duse Adriano G, Jenkins Philip, O'Brien Thomas F, Pablos-Mendez Ariel, Laxminarayan Ramanan (2005). Antimicrobial resistance in developing countries. Part II: strategies for containment. The Lancet Infectious Diseases.

[34] Llewelyn M, et al. The antibiotic course has had its day. BMJ. 2017;358(3418):j3418–23. 10.1136/bmj.j3418 28747365

[35] Horne Rob, Chapman Sarah C. E., Parham Rhian, Freemantle Nick, Forbes Alastair, Cooper Vanessa (2013). Understanding Patients’ Adherence-Related Beliefs about Medicines Prescribed for Long-Term Conditions: A Meta-Analytic Review of the Necessity-Concerns Framework. PLoS ONE.

[36] Blozik E (2012). Telemedicine can help to ensure that patients receive timely medical care. J Telemed Telecare.

[37] Ugur K (2019). Ciprofloxacin is not a better choice in the patients with diabetes suffering urinary tract infection. Dicle Tıp Dergisi.

[38] Kabbani S (2018). Opportunities to Improve Fluoroquinolone Prescribing in the United States for Adult Ambulatory Care Visits. Clin Infect Dis.

