# Supplementary material for: What influences antibiotic sales in rural Bangladesh? A drug dispensers’ perspective
Source: J Pharm Policy Pract. 2020 Jun 3;13:20. doi: 10.1186/s40545-020-00212-8 (PMC7268404; doi:10.1186/s40545-020-00212-8)
Supplement: Supplementary file 1 — Additional file 1. Appendix A. eCRF registry form drug dispenser characteristics (for mapping exercise). Appendix B. eCRF_Dispensers Inventory. Appendix C. Preparatory_Antibiotic Dispensers_In-Depth Interview Guide. [file 40545_2020_212_MOESM1_ESM.docx]

**Appendixes:**

**Appendix A. eCRF registry form drug dispenser characteristics (for mapping exercise)**

Antibiotic dispenser study code: ……_....... [Study site]_[antibiotic dispenser]

Types of Dispensers:

 Hospital pharmacy  Retail pharmacy  Clinic with physician  Clinic without physician  Chemical shop / Drug store  Convenience store / Grocer  Traditional healer  Community health worker  Street vendor (peddler) / Market vendor  Other: ………………………

Funding of the dispensers business:

 Public  Private  Missionary  Unknown

Do the dispensers have Legal / formal authorization?

 Yes  No  Unknown

Expected number of daily antibiotic encounters: …………………….

Do you have plan moving your business location:  Yes  No

If moving location, contact information: ………………………………………………….......

If fixed location, GPS: …………………, ………

**Appendix B. eCRF _ Dispensers Inventory**

1. This is an inventory of the availability and provision quality of 5 essential systemic antibiotics. The example given below concerns amoxicillin; one of the 5 antibiotics inventoried.
2. Tablets, intravenous and intramuscular antibiotics are assessed, not other formula like droplets or creams.
3. For each antibiotic inventoried, the corresponding required storage conditions are processed into the questionnaire (see table below).
4. The temperature and the humidity of the storage sites will be assess using a thermo-hygrometer. Antibiotic (ATC code) Required storage conditions

**Antibiotic Dispenser**

[study site] _ [antibiotic dispenser] …….….........­_..................

Antibiotic name:………………………………………ATC code:………………………………

Name of the brand of Antibiotics : ........…….….................

Please check **X**  the right box

Date of collecting information ............../.............../..........................

Address:......................................................................................

Mobile Number: .....................................

1. Is the medicine available for dispensing at the moment? Yes No

If yes, answer question 2 to 11 for each available brand of the particular antibiotic separately.

If no, proceed to the next antibiotic*.*

2. What is the temperature and humidity at the medicine’s actual storage location?

(measured with a digital thermometer. Note that If the dispenser does not want the field worker to see the storage area, they should be requested to place the thermometer in the relevant part of the storage area themselves, in order to obtain a valid temperature reading. Readings should be taken in the main storage area, not in the part of the shop where they are put on display). …….°C

3. Is the medicine stored protected from light? Yes No

4. What is the humidity at the medicine’s actual storage area?

(*Measurement to be taken using a simple hygrometer, or humidity monitoring device*) ………%

5. Is the medicine packaged in its closed and original blister pack? Yes No

If no, skip to question

6. If yes, is the blister pack packaged in its closed and original box? Yes No

7. Is an expiration date printed on the box? Yes No

8. Is the medicine before its expiration date? Yes No

9. Is the medicine accompanied by a package insert Yes No

*If no, skip to question 11*

10. If yes, is the text of the package insert written in the prevailing language? Yes No

11. Write down the following information about the available brands of the medicine sold by the dispenser in the table below:

The following questions are about antibiotics sold by the dispenser (Circle only one answer for each question)

| Medicine brand | Dose  (mg) | Quantity | Formulation  (capsule/Tablet/suspension/intervenus/ intramuscular) | Price  (local currency) | Country of Manufacture |
| --- | --- | --- | --- | --- | --- |
|  |  |  |  |  |  |

12. Are antibiotics supplied exclusively with a prescription? Yes No Variable

13. Are antibiotics supplied in their closed and original blister pack? Yes No Variable

14. Are blister packs supplied in their closed and original box? Yes No Variable

15. Are antibiotics supplied with written instructions for use? Yes No Variable

16. Are antibiotics supplied with verbal instructions for use? Yes No Variable

We have finished the inventory. Thank you for your participation.

Name of interviewer: **...................................**

Signature of interviewer: **...................................**

**Appendix C. Preparatory _Antibiotic Dispensers _In-Depth Interview Guide**

We need to be sure that we include the full range of the different types of antibiotic dispensers in the sample (licensed and otherwise), as identified in the mapping exercise in each HDSS. We should also include in the sample employees who work directly with customers.

In order to ensure that these interviews produce good insights, the interviewers will need to have a good background understanding of their country’s regulations on antibiotic sales. This will need to be included in their training.

**Demographic Information of respondent**:

Name, Age, sex, Education level, location and type of business, length of time in the business total and at current business, position in business.

***The medicines:***

1. Please tell me briefly about the range of different medicines that you sell. Which are the popular ones?

2. What is/are the source/s of the various medicines that you sell? Do your dispensers bring the medicines to your shop, or do you pick them up yourselves from your dispensers?

3. What informs your decision to buy from the dispensers that you have mentioned? (Probe for details of quality, cost, credit facilities, packaging, incentives, etc).

4. What are your experiences with getting your supply of different medicines? Probe regarding availability/shortages etc., for different categories of medicines:

(A. Anti-malarial, B. Painkillers, C. Cough medicines , D. Antibiotics, E. Haematinics , F. Any others)

5. Where do you think the medicines you sell are manufactured? What are your impressions about the quality of the medicines from different origins? Are certain origins more popular than others?

6. From where do you receive information/learn about the different medicines that you sell? [Probes: Do you feel you have enough information, or are there certain medicines that you would like to know more about? What would be the best way for you to learn about these medicines?]

7. Which medicines do you usually sell to people who have coughs and colds? What determines the length of the course that you supply?

8. What do you know about antibiotics? Explain. What proportion of all the medicines that you sell are antibiotics? Which are your five most commonly sold antibiotics?

9. For which illnesses do you usually sell antibiotics? What determines the choice of antibiotic?

10. How and where do you store your antibiotics?

11. Do you check the expiry dates on the antibiotics you sell? What do you do with drugs that have passed their expiry date?

12. Do customers ever bring back unused drugs? If so, what do you do with them?

***The customers:***

13. Can you describe one of your recent dispenses for me? How does this go?

14. Do many of your customers ask for particular medicines without any prescription (either through self-medication, or on verbal recommendation of health workers)? Is there a certain sort of customer who does this (e.g. male, female, young, old), and what are the most common conditions that they treat on this basis? Do they ask specifically for antibiotics?

15. Do you ever feel encouraged to sell your customers antibiotics that you think maybe they don’t need? If so, is this encouragement perhaps related to your customers or to your dispensers? How do you respond to such situations? Details.

16. Do you ever give any sort of information (verbal or written) to your customers about the antibiotics that you sell them? What information do you give? Do they ever ask questions? Examples. [Probe: do you ever ask about allergies?

17. Do your customers ever voice any concerns about the various medicines you sell? Details?

18. Are you aware of any medicine sellers in this area who either sell incomplete doses of antibiotics [probe: if, for example, a customer doesn’t have money for the whole dose. Or for any other reason.], or who sell more antibiotics than are needed [probe: for future use]? Is it common for you to dispense a mix of antibiotics with other medicines? Are these practices generally seen as acceptable or not? Details.

19. Do you think that your customers sometimes fail to take their full course of antibiotic treatment? If so, why do you think this is?

20. Do you ask your customers if they have used antibiotics before and for what conditions/diseases, in particular when they are requesting a specific antibiotic?

***Antibiotic resistance:***

21. Do you think you have a good understanding of what antibiotic resistance is, how it is caused, and what its implications are? Is there anything on this topic that you would like to know more about? Details.

22. What do you think would be the best way to inform people (both medicine sellers and the community) about proper antibiotic use and the dangers of antibiotic resistance?

23. Overall, what do you think should be done to improve appropriate antibiotic use and decrease antibiotic resistance in this country?

***Regulatory issues:***

24. What are the regulations that you have to follow in order to sell antibiotics [probe regarding prescriptions]? What challenges, if any, do you face in following these regulations?

25. Do you think that most sellers know the regulations regarding selling antibiotics? If so, do they always follow them? If not, why not? Do you know whether these regulations are being checked or audited by authorities?

26. Are there any penalties for sellers who sell antibiotics without prescription? Details.

27. Do you think current government regulations are sufficient to control inappropriate antibiotic use? If not, how could things be improved?

28. What would happen to your business if antibiotic sales declined due to closer compliance with regulations on dispensing antibiotics? Would this be a problem for you or your business? If yes, how? What would you suggest as a means of resolving this problem?

We have finished the interview. Thank you for your participation.
